# Supplementary material for: Splice-Junction-Based Mapping of Alternative Isoforms in the Human Proteome
Source: Cell Rep. Author manuscript; Available in PMC 2020 Jan 15. (PMC6961840; doi:10.1016/j.celrep.2019.11.026)
Supplement: 2 [file NIHMS1546469-supplement-2.zip › DF1/PXD010154/AdrenalGland/AdrenalGland_7_UBXN11_PGPGPGPSPGPGPGPSPCPGPSPSPR.pdf]

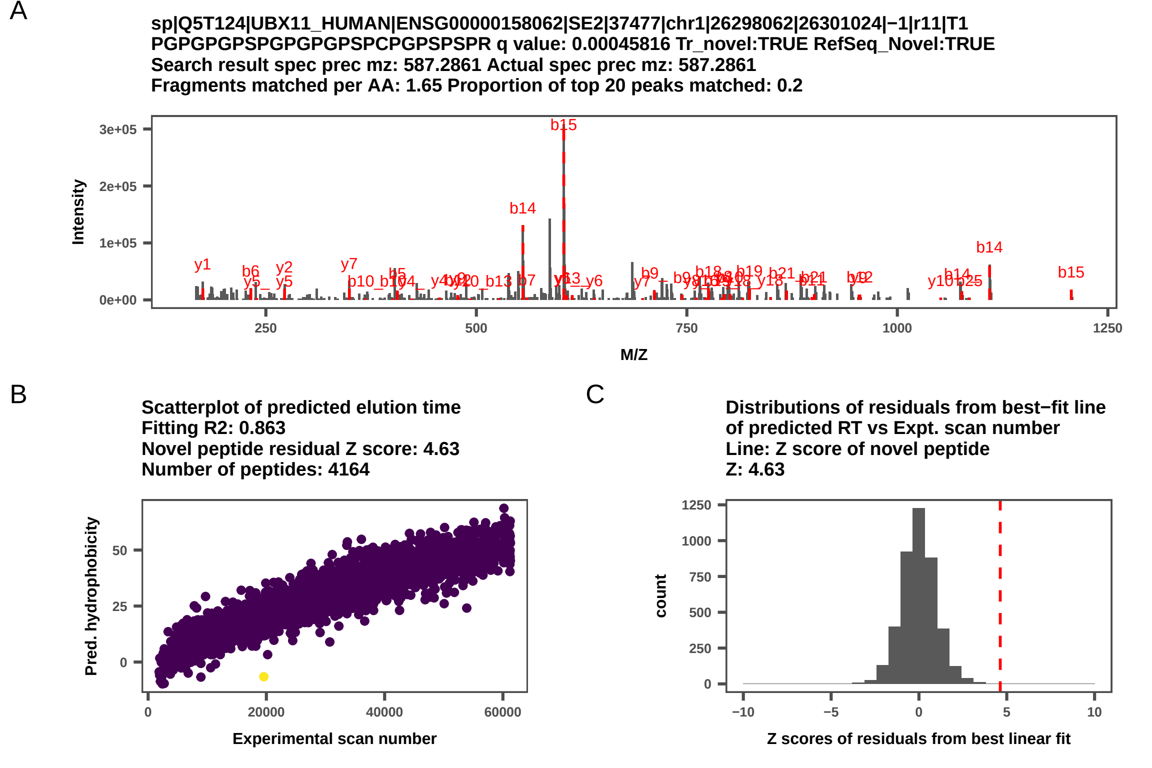
A
sp|Q5T124|UBX11_HUMAN|ENSG00000158062|SE2|37477|chr1|26298062|26301024|−1|r11|T1
PGPGPGPSPGPGPGPSPCPGPSPSPR q value: 0.00045816 Tr_novel:TRUE RefSeq_Novel:TRUE
Search result spec prec mz: 587.2861 Actual spec prec mz: 587.2861
Fragments matched per AA: 1.65 Proportion of top 20 peaks matched: 0.2
Intensity
3e+05
2e+05
1e+05
0e+00
b15
b14
y1
b6
y2
y5
y7
b14
b15
250
500
750
1000
1250
M/Z
B
Scatterplot of predicted elution time
Fitting R2: 0.863
Novel peptide residual Z score: 4.63
Number of peptides: 4164
Pred. hydrophobicity
50
25
0
0
20000
40000
60000
Experimental scan number
C
Distributions of residuals from best−fit line
of predicted RT vs Expt. scan number
Line: Z score of novel peptide
Z: 4.63
count
1250
1000
750
500
250
0
−10
−5
0
5
10
Z scores of residuals from best linear fit
